# NQO1 C609T polymorphism and esophageal cancer risk: a HuGE review and meta-analysis

**DOI:** 10.1186/1471-2350-14-31

**Published:** 2013-03-05

**Authors:** Hu Yanling, Zhang Yuhong, He Wenwu, Xian Lei, Chen Mingwu

**Affiliations:** 1 Medical Research Center of Guangxi Medical University, Nanning, Guangxi, China; 2 Department of gastro enterology, First Affiliated Hospital, Guangxi Medical University, Nanning, Guangxi, China; 3 Department of Cardiothoracic Surgery, First Affiliated Hospital, Guangxi Medical University, Nanning, Guangxi, China; 4 Department of Cardiothoracic Surgery, Nanchong Central Hospital • The Second Clinical College of North Sichuan Medical college, Nanchong, Sichuan, China

## Abstract

**Background:**

Many studies have been carried out to test the hypothesis that the NQO1 C609T polymorphism might be associated with the risk of esophageal cancer. However, the results are poorly consistent, partly due to genetic or other sources of heterogeneity. To investigate the association between this polymorphism and the risk of esophageal cancer, a meta-analysis was performed.

**Methods:**

We used odds ratios (ORs) with 95% confidence intervals (CIs) to assess the strength of association. The frequency of the putative risk allele in the controls was estimated by the inverse-variance method. Cochran’s Q statistic and the inconsistency index (I^2^) were used to check heterogeneity. Egger’s test and an inverted funnel plot were used to assess the publication bias.

**Results:**

Our study included eight published case-control studies about the NQO1 C609T polymorphism and esophageal cancer, including a total of 1,217 esophageal cancer patients and 1,560 controls. Overall, a significant association was found between the NQO1 C609T variant and esophageal cancer under a recessive model (OR = 1.647; 95% CI = 1.233-2.200). Regarding histological type, more significant evidence was found for esophageal squamous cell carcinoma (ESCC) (OR = 2.03; 95% CI = 1.29-3.19) than esophageal adenocarcinoma (EAC) (OR = 1.61; 95% CI = 1.01-2.56) under a recessive model.

**Conclusions:**

The meta-analysis suggests that the NQO1 C609T polymorphism considerably increases the risk of esophageal cancer.

## Background

Esophageal cancer is a malignancy of the esophagus, the muscular tube through which food passes from the throat to the stomach. Esophageal tumors usually lead to dysphagia, pain and other symptoms, and are diagnosed by biopsy. Generally, esophageal cancer has two subtypes, squamous cell cancer (ESCC) and adenocarcinoma (EAC). Squamous cell cancer arises from the cells that line the upper part of the esophagus. Adenocarcinoma arises from glandular cells that are present at the junction of the esophagus and the stomach [1].

NAD(P)H quinone oxidoreductase 1 (NQO1) is a member of the NAD(P)H dehydrogenase (quinone) family and encodes a cytoplasmic 2-electron reductase, which is a cytosolic flavoenzyme that protects cells from oxidative damage [2]. NQO1 catalyzes the reductive activation of quinoid chemotherapeutic agents and environmental carcinogens such as heterocyclic amines, nitrosamines and cigarette smoke condensates [3]. The NQO1 T allele has only 2 to 4% enzymatic activity in comparison to its wild type form. Cells homozygous for the polymorphic NQO1 allele (T/T) express NQO1 mRNA, but not the protein because the mutant NQO1 protein is rapidly degraded by the proteasomal system [4]. However, the activity of the NQO1 enzyme may be influenced by a major polymorphism involving a single C to T substitution at nucleotide 609 of exon 6 in the NQO1 cDNA that causes a Pro187Ser amino acid change [5].

Some studies have shown that this polymorphism in the NQO1 gene affects the translation of the NQO1 protein. Compared to the homozygous wild type (C/C), expression of the NQO1 protein encoded by the heterozygous phenotype (C/T) is decreased approximately three-fold. In addition, the homozygous mutant (T/T) phenotype causes a complete lack of enzyme activity [3,5-7]. The NQO1 C609T polymorphism has been associated with the risk of various cancers such as renal [8], lung [9,10], esophageal [11-13], colorectal [14], and head and neck [15]. However, the results of some studies on the effect of the NQO1 C609T polymorphism on esophageal cancer are debatable. Meta-analyses are usually useful when many studies point more or less in the same direction, but a single study does not have sufficient power to show a significant result. Therefore, a meta-analysis of these studies was undertaken to investigate the association of the NQO1 C609T polymorphism with susceptibility to esophageal cancer.

## Methods

### Search strategy and data extraction

All original studies published in English on the NQO1 C609T polymorphism and esophageal cancer were considered in our meta-analysis. HuGENet, Embase and PubMed were searched up to August 8 2011, using the following terms: (“esophageal cancer” or “esophagus” or “ESCC” or “EAC” or “oesophagus”) and (“polymorphism” or “SNP” or “allele” or “variant”) and (“NQO1” or “NAD(P)H: quinine oxidoreductase 1” or “NAD(P)H dehydrogenase, quinone 1” or “DHQU”).

Included studies had to fit the following criteria: (1) sufficient data regarding allele frequency; (2) an association analysis between the NQO1 C609T polymorphism and esophageal cancer risk; and (3) independent case-control studies.

### Data extraction

The following information was extracted by three investigators (Y-L H, W-W H and Y-H Z) from each study: the first author, year of publication, country, race, sample size, outcome, characteristics of controls, case and control diagnostic criteria, genotyping method, allele frequencies, genotype distribution in cases and controls, pathology status of esophageal cancer and the esophageal cancer risk factors. Results were compared and different opinions were resolved by a discussion.

### Statistical analysis

Hardy-Weinberg equilibrium (HWE; P ≥ 1e-03) and chi-square test methods were used to test the distribution of genotypes in the control group of each study. The frequency of the putative risk allele in the controls was estimated by the inverse-variance method [16-18]. Cochran’s Q statistic and the inconsistency index (I^2^) were used to check heterogeneity [19]; if P > 0.10 and I^2^ < 25%, heterogeneity did not exist among the studies [20]. If there was no heterogeneity, logistic regression with fixed effects was used to evaluate the overall gene effect; otherwise, the random-effects model was used. To determine the overall gene effect, the model that included the gene was compared with the model that did not. If the overall gene effect was statistically significant, further comparisons of OR1 (AA vs. aa), OR2 (Aa vs. aa) and OR3 (AA vs. Aa) were explored with A as the risk allele. We selected the genetic models according to the following criteria [20]:

If OR1 = OR2 ≠ 1 and OR3 = 1, the dominant model was selected.

If OR1 = OR3 ≠ 1 and OR2 = 1, the recessive model was accepted.

If OR2 = 1/OR3 ≠ 1 and OR1 = 1, the overdominant model was taken.

If OR1 > OR2 > 1 and OR1 > OR3 > 1(or OR1 < OR2 < 1 and OR1 < OR3 < 1), the codominant model was adopted. Finally, the results were pooled again under the appropriate genetic model.

Egger’s test and an inverted funnel plot was used to assess publication bias [21]. HWE was checked in the control group of the eligible studies by the chi-square test (p ≤ 0.001). Sensitivity analysis was performed including studies that deviated from HWE. Statistical tests were performed using the STATA software, version 11.1 (Stata Corporation, USA). All P values were two-sided.

## Results

### Study inclusion and characteristics

Ten relevant studies describing the association between NQO1 C609T and esophageal cancer were identified. However, after reading the full text, we excluded two of these ten studies due to overlapping [22,23]. Finally, eight studies met the inclusion criteria and were included [24-31]. Among these, five were on Caucasians [24,26-29] and three on Asians [25,30,31]. All the included studies were case-controlled, comprising 1,217 cases and 1,560 controls.

Among the included articles described in Table 1, four selected esophageal cancer patients based on endoscopy or histological diagnosis [24-26,31], while the other four [27-30] selected cases that underwent esophagectomy without prior radio- and/or chemotherapy. All the controls were recruited in the same period as the cases. However, the source of the controls for each study was not similar. In the included studies, five studies [27-31] selected healthy individuals as controls, while three [24-26] chose hospital patients as controls.

**Table 1:** The studies summary of NQO1 C609T polymorphism with esophageal cancer

**Ref.**	**Investigator**	**Year**	**Country**	**Race**	**Eligible subjects**				**Characteristic**		**Source of controls**	**Method**
					**Cases**	**Controls**	**ESCC**	**EAC**	**Cases**	**Controls**		
[25]	Hamajima et al.	2002	Japan	Asian	102	241	*	*	The patients were invited to participate in the present study by doctors in charge. They were enrolled between March 1999 and December 2000 at Aichi Cancer Center Hospital.	Controls were sampled from patients at Aichi Cancer Center Hospital during the same period as for the cases; participants in a Helicobacter pylori eradication program without a history of cancer who underwent gastroscopy.	Hospital-based	PCR-CTPP
[27]	Sarbia et al.	2003	Germany	Caucasian	61	252	0	61	Patients who underwent oesophagectomy for oesophageal adenocarcinoma between 1987 and 2001.	Healthy blood donors of the Heinrich Heine University Blood Donation Centre between 1995 and 2001. They were from the same geographic region as case groups but were unrelated.	Population -based	PCR-RFLP
[29]	Zhang JH et al.	2003	Germany	Caucasian	257	252	257	0	All ESCC patients that underwent esophagectomy without prior radio- and/or chemotherapy between 1978 and 1998 in the Department of Surgery of the Heinrich Heine University, Duesseldorf.	The healthy controls from the German Caucasian population were unrelated blood donors from the same region as the ESCC patients.	Population-based	PCR-RFLP
[30]	Zhang JH et al.	2003	China	Asian	317	306	193	124	All ESCC patients that underwent esophagectomy without prior radio- and/or chemotherapy between 2001 and 2002 in the Fourth Affiliated Hospital, Hebei Medical University.	The healthy controls from the northern Chinese population were unrelated blood donors from the same region as the ESCC patients.	Population-based	PCR-RFLP
[28]	Rahden et al.	2005	Germany	Caucasian	140	260	0	140	The patients that underwent esophagectomy without prior radio- or chemotherapy between 1991–2003 at the Technical University of Munich.	Healthy volunteers subjects hospitalized for traumatic injuries but without any history of cancer.	Hospital-based	PCR
[31]	Zhang WC et al.	2006	China	Asian	106	106	*	*	The patients were diagnosed for primary esophageal cancer by pathology or endoscopy between 2003–2004, they were Han Chinese person.	The healthy controls from the same region without digestive system disease and any history of cancer.	Hospital-based	PCR-RFLP and ASPCR
[24]	Martino et al.	2007	UK	Caucasian	141	93	0	141	The patients were diagnosed based on endoscopic and histological evidence.	Control individuals had been recruited from a dyspepsia endoscopy list, 44 of these (47%) reported reflux-related symptoms, such as heartburn and/or regurgitation.	Hospital-based	PCR-RFLP
[26]	Marjani et al.	2010	Iran	Caucasian	93	50	93	0	The criteria for enrollment patients were an age of at least 18 years and be resident of the study area at registration time, with no concurrent or previous history of other cancer in any organ.	Controls with no malignancy and/or severe diseases, were enrolled between the years 2002 and 2008, with the same age and residence criteria as for the cases.	Hospital-based	PCR-RFLP

Annotation: The “*” representative the study samples have not be classified to EAC or ESCC.

In addition, among these eligible studies, two included populations that were selected from ESCC patients [26,29], four from EAC patients [24,27,28,30], while the other two did not clarify the histological type [25,31].

### Meta-analysis database

Overall, the eligible studies included 1,124 cases and 1,510 controls that were genotyped. The prevalence rates of TT in C609T variants were 19.1% in the controls of Asian descent and 4.1% in the controls of Caucasian descent. The prevalence rates of CT for controls of Caucasian and Asian decent were 45.5% and 31.7%, respectively. For histological type, the prevalence rates of TT were 8.6% and 7.3% in controls of ESCC and EAC patients, respectively, while CT were 40.9% and 34.0% in controls of ESCC and EAC patients, respectively. The genotype distribution and all the P-values for HWE testing are shown in Tables 2 and 3.

**Table 2:** Frequency of NQO1 C609T polymorphism in different populations included in a meta-analysis

**Ref.**	**Investigator**	**Year**	**Sample size (%)**	**Race**	**Cases (%)**			**Controls (%)**			**P value for HWE**
					**CC**	**CT**	**TT**	**CC**	**CT**	**TT**	
[25]	Hamajima et al.	2002	343(10.47)	Asian	36.3	51.0	12.7	35.7	44.4	19.9	0.165
[27]	Sarbia et al.	2003	313(9.55)	Caucasian	49.2	47.5	3.3	73.4	25.0	1.6	0.602
[29]	Zhang JH et al.	2003	509(15.54)	Caucasian	71.2	21.8	7.0	73.4	25.0	1.6	0.602
[29]	Zhang JH et al.	2003	499(15.23)	Asian	26.4	47.7	25.9	34.0	49.6	16.3	0.765
[30]	Zhang JH et al.	2003	623(19.02)	Asian	32.3	44.4	23.4	31.5	52.1	16.4	0.390
[28]	Rahden et al.	2005	400(12.21)	Caucasian	65.0	30.0	5.0	71.2	25.0	3.8	0.166
[31]	Zhang WC et al.	2006	212(6.47)	Asian	26.4	46.2	27.4	40.6	35.8	23.6	0.007
[24]	Martino et al.	2007	234(7.14)	Caucasian	68.1	30.5	1.4	59.1	35.5	5.4	0.986
[26]	Marjani et al.	2010	143(4.37)	Caucasian	54.8	37.6	7.5	44.0	48.0	8.0	0.467

**Table 3:** Frequency of NQO1 C609T polymorphism in ESCC and EAC patients included in a meta-analysis

**Ref.**	**Investigator**	**Year**	**Histological type**	**Cases (%)**			**Controls (%)**			**P value for HWE**
				**CC**	**CT**	**TT**	**CC**	**CT**	**TT**	
[27]	Sarbia et al.	2003	EAC	49.2	47.5	3.3	73.4	25.0	1.6	0.602
[29]	Zhang et al.	2003	ESCC	71.2	21.8	7.0	73.4	25.0	1.6	0.602
[29]	Zhang et al.	2003	ESCC	26.4	47.7	25.9	34.0	49.6	16.3	0.765
[30]	Zhang et al.	2003	EAC	32.3	44.4	23.4	31.5	52.1	16.4	0.390
[28]	Rahden et al.	2005	EAC	65.0	30.0	5.0	71.2	25.0	3.8	0.166
[26]	Marjani et al.	2010	ESCC	54.8	37.6	7.5	44.0	48.0	8.0	0.467

### Main results, subgroup analyses

#### NQO1 C609T polymorphism and ethnic group

The results of the included studies regarding the association between the NQO1 C609T polymorphism and esophageal cancer were conflicting, as seen in Table 1. Hamajima et al [25], Rahden et al [28], Zhang et al [31] and Marjani et al [26] showed no significant association with esophageal cancer. However, the other four studies showed a significant association with esophageal cancer [24,27,29,30]. All the included studies were in HWE.

After sensitivity analysis, Hamajima et al [25] and Martino et al [24] had the highest sensitivity, and were removed because the controls included no healthy people, which was determined by reading the full text. After removing these two articles, the phenotyped samples contained 974 cases and 1,226 controls.

Using the inverse variance fixed effects model, the ORs of the pooled NQO1 T allele frequencies were 1.32 (95% CI: 1.08-1.62) for Caucasians and 1.32 (95% CI: 1.08-1.60) for Asians. In total, the summary OR for all the studies was 1.32 (95% CI: 1.15-1.52). It was shown that the T allele was related to susceptibility to esophageal cancer.

**Figure 1:**
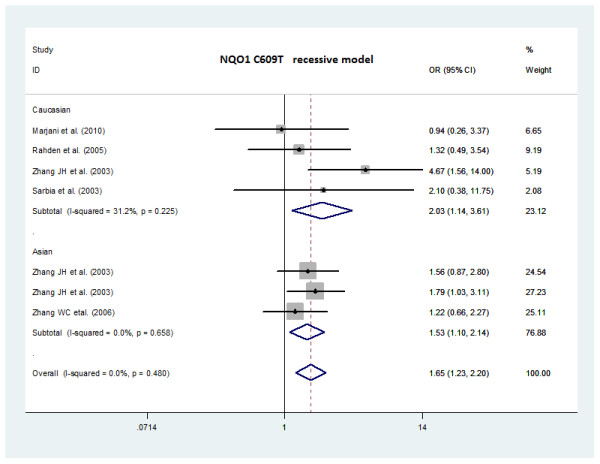
**The forest plot describing the meta- analysis with a fixed-effect recessive model (TT versus CT + CC) for the association of NQO1 C609T polymorphism with esophageal cancer. **Each study is depicted with size inversely proportional to its variance, accompanied by the respective 95% confidence intervals. Values of OR > 1, implied an increased risk for esophageal cancer with the TT genotype.

According to the principle of genetic model selection by Thakkinstian [17], the recessive model was determined. The summary result showed a significant relationship between the NQO1 C609T polymorphism and esophageal cancer (Figure 1) from the meta-analysis of the phenotype studies. For the recessive model, the overall pooled odds ratio using a fixed effect model was 1.65 (95% CI: 1.23-2.20). The ORs for Caucasians and Asians were 2.03 (95% CI:1.14-3.61) and 1.53 (95% CI:1.10-2.14), respectively. Through heterogeneity analysis, we found no evidence for heterogeneity among the studies (for the recessive model, I^2^ = 0.0%, P = 0.48). In addition, Egger’s test showed that publication bias was not significant under the recessive model (P = 0.69).

### NQO1 C609T polymorphism and histological type of esophageal cancer

Six case-control articles reporting an association between the NQO1 C609T polymorphism and the histological type of esophageal cancer were included in our research, but their results were debatable. For EAC, Martino et al [24] and Zhang et al [30] showed a significant association, but Rahden et al [28] showed no significant correlation. For ESCC, Sarbia et al [27] and Zhang et al [29] showed a significant association, but Marjani et al [26] did not. After removing this study, the samples of different histological types contained 868 cases and 1,120 controls.

**Figure 2:**
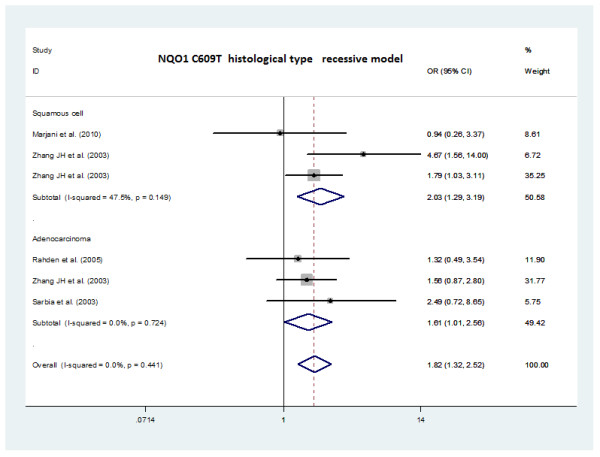
**The forest plot describing the meta- analysis with a fixed-effect recessive model (TT versus CT + CC) for the association of NQO1 C609T polymorphism with ESCC and EAC. **Each study is depicted with size inversely proportional to its variance, accompanied by the respective 95% confidence intervals. Values of OR > 1, implied an increased risk for esophageal cancer with the TT genotype.

The recessive model was selected according to the principle of Thakkinstian [17]. The summary result from the meta-analysis of the phenotype studies indicated a significant relationship between the NQO1 C609T polymorphism and the histological type of esophageal cancer (Figure 2). For the recessive model, the overall pooled odds ratio using the fixed effect model was 1.82 (95% CI: 1.32-2.52). The ORs of ESCC and EAC were 2.03 (95% CI:1.29-3.19) and 1.61 (95% CI:1.01-2.56), respectively. Through heterogeneity analysis, we found no evidence for heterogeneity among the studies (for the recessive model, I^2^ = 0.0%, P = 0.44). Finally, Egger’s test showed that publication bias was not significant under the recessive model (P = 0.74).

To test whether heterogeneity of the samples affected the results of the meta-analysis, we performed additional analysis. We only used the samples from Germany and China to test the ethnicity effect. The ORs of Germany and China were 1.68 (95% CI: 1.13-2.51) and 1.96 (95% CI: 1.36-2.82), respectively. There was no obvious difference in the results from Caucasian and Asian patients.

## Discussion

NQO1 acts as an imperative part of the cellular antioxidant defense system by detoxifying quinines and can prevent the formation of reactive oxygen species. NQO1 gene mutations are linked to tardive dyskinesia, which increases the risk of hematotoxicity after exposure to benzene, and susceptibility to various forms of cancer. Many studies have been carried out to test the hypothesis that the NQO1 C609T polymorphism might be linked to the risk of esophageal cancer, but the results are controversial. This meta-analysis, involving a total of 1,217 esophageal cancer patients and 1,560 controls from eight case-control studies, examined the association of one polymorphisms of the NQO1 gene with esophageal cancer risk. The OR of the pooled NQO1 T allele frequencies for all the studies was 1.32 (95% CI: 1.15-1.52). In addition, significant evidence was found for a correlation between the NQO1 C609T variant and esophageal cancer under the recessive model (OR = 1.647; 95%CI = 1.233-2.200). Similar to our study, other reports have also shown an association of NQO1 609C > T polymorphism with susceptibility to esophageal tumors. Marjani et al [26] observed that NQO1 expression in esophageal tumor tissue was related to the NOQ1C609T genotype, with its expression elevated when the T allele appeared in the NOQ1C609T genotype.

Different enzymes encoded by varying NQO1genotypes may affect enzyme activities, and the lack of NQO1 activity encoded by the homozygous TT genotype might result in reduced detoxification of exogenous carcinogens. Traver et al [32] confirmed that the NQO1 homozygous variant (TT) had very little or no quinine reductase activity; however, compared to the homozygote (CC), the heterozygote variant (CT) showed approximately one-third of the enzymatic activity. Recent studies have identified a C➙ T mutation in NQO1 [33]. Two to 4% of the global human population carry both mutant alleles and are deficient in NQO1. Mice lacking NQO1 gene expression accumulate lower amounts of abdominal fat and show increased sensitivity to menadione-induced hepatic toxicity compared to wild type mice [11,12]. Delwin et al [13] showed that loss of NQO1 caused mycloid hyperplasia of the bone marrow and significant increases in blood neutrophils, eosinophils and basophils in NQO1-null mice.

A heterogeneity evaluation is always conducted in statistical analysis in meta-analysis. However, we found low statistical power during heterogeneity testing. Therefore, several subgroup meta-analyses were performed according to ethnicity, control source and case classification. In the racial subgroups, there was a statistically significant asssociation between the NQO1 polymorphism and esophageal cancer. The ORs for Caucasians and Asians were 2.03 (95% CI:1.14-3.61) and 1.53 (95% CI:1.10-2.14), respectively. This showed a possible role of ethnic divergence. Wang et al [34] also noted that the overall frequency of the NQO1 609 T allele was nearly double among Asians than in Europeans in both patients and controls. There is a wide ethnic variation in allelic frequencies of NQO1 609C > T according to the Hapmap database (http://www.hapmap.ncbi.nlm.nih.gov): 18.6% in Europeans (CEU) and 50% in Asian populations (HCB).

The results of this meta-analysis should be interpreted with some caution because there were limitations in our analysis. First, selection bias was a possible major source of heterogeneity from uncontrolled confounders and bias inherent in the study design. For example, there was not an identical criterion for cases and controls among all the included articles. Second, different genotype ratios may have a potential impact on the outcomes. The rate of the TT genotype was very low in Caucasian subjects (fewer than 10% of all Caucasian studies), but showed a moderate distribution in Asians (more than 15% in all Asian studies). Third, we only considered the NQO1 C609T polymorphism in esophageal cancer. However, there may be a possible interaction between the NQO1 C609T polymorphism and other environmental factors.

## Conclusions

In summary, the present meta-analysis provides information that the NQO1 C609T polymorphism considerably increases susceptibility to or prognosis of esophageal cancer, especially in ESCC patients. Additional population-based studies in large sample sizes should be conducted before its clinical application.

## Competing interests

The authors declare that they have no competing interests.

## Authors’ contributions

YH collected the literature data, developed the statistical model, carried out the software implementation and drafted the manuscript. WH collected the literature data, read the full text articles, and checked the model and results. YZ collected the literature data, read the full text articles and drafted the manuscript. XL helped with the discussion in theoretical developments, as well as in drafting the manuscript. MC helped with the discussion both in the theoretical development and English copyediting. All authors read and approved the final manuscript.

## Pre-publication history

The pre-publication history for this paper can be accessed here:

http://www.biomedcentral.com/1471-2350/14/31/prepub
